# Papillomavirus pseudovirions packaged with the L2 gene induce cross-neutralizing antibodies

**DOI:** 10.1186/1479-5876-8-28

**Published:** 2010-03-24

**Authors:** Nicolas Combelas, Emilie Saussereau, Maxime JJ Fleury, Tatiana Ribeiro, Julien Gaitan, Diego F Duarte-Forero, Pierre Coursaget, Antoine Touzé

**Affiliations:** 1 Inserm U618 "Protéases et vectorisation pulmonaires", Tours; University François Rabelais, Tours, France and IFR 136 "Agents Transmissibles et Infectiologie", Tours, France; 2 Instituto Nacional de Cancerologia, Bogotà, Colombia; 3 Current address: EA 3855 Microenvironnement de l'Hématopoïèse et Cellules Souches, University François Rabelais, Tours, France

## Abstract

**Background:**

Current vaccines against HPVs are constituted of L1 protein self-assembled into virus-like particles (VLPs) and they have been shown to protect against natural HPV16 and HPV18 infections and associated lesions. In addition, limited cross-protection has been observed against closely related types. Immunization with L2 protein in animal models has been shown to provide cross-protection against distant papillomavirus types, suggesting that the L2 protein contains cross-neutralizing epitopes. However, vaccination with L2 protein or L2 peptides does not induce high titers of anti-L2 antibodies. In order to develop a vaccine with the potential to protect against other high-risk HPV types, we have produced HPV58 pseudovirions encoding the HPV31 L2 protein and compared their capacity to induce cross-neutralizing antibodies with that of HPV L1 and HPV L1/L2 VLPs.

**Methods:**

The titers of neutralizing antibodies against HPV16, HPV18, HPV31 and HPV58 induced in Balb/c mice were compared after immunization with L2-containing vaccines.

**Results:**

Low titers of cross-neutralizing antibodies were detected in mice when immunized with L1/L2 VLPs, and the highest levels of cross-neutralizing antibodies were observed in mice immunized with HPV 58 L1/L2 pseudovirions encoding the HPV 31 L2 protein.

**Conclusions:**

The results obtained indicate that high levels of cross-neutralizing antibodies are only observed after immunization with pseudovirions encoding the L2 protein. HPV pseudovirions thus represent a possible new strategy for the generation of a broad-spectrum vaccine to protect against high-risk HPVs and associated neoplasia.

## Background

The fact that cervical cancer is the second most common cause of cancer deaths in women worldwide [1], and that virtually all cervical cancers are etiologically linked with infection by "high risk" human papillomavirus (HPV) [2], has encouraged the development of prophylactic vaccines to prevent genital infection. Fifteen of the HPV types infecting the mucosal epithelium cause cervical cancer, HPV16 and 18 being the most prevalent types detected in cervical carcinoma [1]. Papillomaviruses are small non-enveloped DNA viruses and their icosahedral capsid is constituted of L1 and L2 proteins, which encapsidate a closed circular, double-stranded DNA of about 8 kbp. The viral capsid of 50-60 nm in diameter contains 72 pentamers of L1 major protein and 12 to 72 copies of L2 minor capsid protein [3,4].

Immunization with L1 self-assembled into virus-like particles (VLPs) induces high titers of neutralizing antibodies and confers protection in animals against homologous experimental infection [5,6]. It has also been shown that protection is mediated by neutralizing antibodies directed against conformational epitopes. These results have led to the industrial development of vaccines against genital HPV types. Pre-clinical studies have shown that the neutralizing antibodies induced by L1 VLPs are predominantly type-specific [7,8]. However, low levels of cross-neutralization have been reported between HPV6 and 11 and HPV 16 and 31 [9-12] and higher levels between HPV18 and 45 [13]. Clinical trials have shown that the immune response is associated with protection against HPV16 and HPV18 infections and associated lesions [14,15].

Current HPV vaccines containing L1 VLPs promote the generation of a strong, mainly type-specific, neutralizing antibody response. Clinical trials with HPV16 and 18 vaccines have also revealed that cross-protection against HPV types is limited to closely related types. Protection against HPV31 lesions was clearly established for both vaccines and protection against HPV45 lesions for only one vaccine [15,16]. As the licensed HPV vaccines target only two of the 15 high-risk HPV, one strategy is to combine many types of L1 VLPs to prevent infection against multiple high-risk types. To address this issue, a multivalent VLP vaccine is currently under clinical trial [17]. However, the inclusion of numerous VLP types complicates vaccine development and would increase the risk of antigenic competition that could result in lower protective efficacy and/or affect long lasting protection against certain HPV types.

The minor capsid L2 protein has emerged as another candidate prophylactic vaccine, since immunization with L2 in animal models of papillomavirus infection induces cross-neutralizing antibodies that are able to mediate broader protection than L1 VLPs [7,18-24]. Preclinical and clinical findings [25-27] have confirmed that L2 vaccines induce broad-spectrum cross-neutralizing antibodies. However, L2 protein and L2 peptides are less immunogenic than L1 VLPs, and it has been reported that the incorporation of the L2 protein into L1 VLPs does not increase the anti-L2 response due to the immunodominance of L1 [23]. This suggests that new vaccine strategies have to be investigated if such an L2-based vaccine is to be effective.

Although most investigations concerning VLPs have dealt with vaccine development, it has also been demonstrated that HPV VLPs can be used to generate pseudovirions (PsV) by packaging unrelated plasmids within the VLPs, and they thus represent a valuable gene delivery system that could be used to induce an immune response against the packaged *de novo *synthesized transgene product [28,29].

The aims of this study were to investigate the possibility of generating an HPV vaccine by packaging a plasmid encoding the HPV 31 L2 protein within HPV58 L1/L2 PsV (PsV58-31L2). The L2-pseudovirion vaccination strategy aims to induce high-titers of conformation-dependent antibodies to L1 similar to those observed with monovalent HPV VLP L1 vaccines and to induce *de novo *L2 expression for augmented immunogenicity to L2 protein in order to cross-neutralize multiple HPV types [30].

## Materials and methods

### Antibodies and Cell lines

CamVir-1 monoclonal antibody (MAb) (BD Biosciences, Le Pont de Claix, France) binds to a linear epitope which has been mapped between amino acids 203 to 209 of the HPV-16 L1 protein [31]. Rabbit anti-HPV16 L2 immune serum was kindly provided by Richard Roden. COS-7 cells (African green monkey kidney cells, ATCC CRL-1651) were grown in Dulbecco's modified Eagle's Medium (Invitrogen, Illkirch, France) supplemented with 10% heat-inactivated fetal calf serum (FCS), 100 IU/ml penicillin, and 100 μg/ml streptomycin and 1 mM sodium pyruvate. The 293FT cell line (Invitrogen) is a fast growing variant of the 293 cell line that stably expresses SV40 TAg and the neomycin resistance gene from pCMVPORT6AT.neo plasmid. 293FT cells were grown in Dulbecco's modified Eagle's Medium, supplemented as above, plus 1% non-essential amino acids and 500 μg/ml G418 (Invitrogen). Cell lines were grown at 37°C in a humidified atmosphere with 5% CO_2_.

### Production of HPV VLP vaccines

HPV31 L1 and HPV31 L1/L2 VLPs were produced and purified from *Sf*21 insect cells infected with recombinant baculoviruses encoding both L1 and L2 proteins as previously described [32,33]. HPV58 L1/L2 PsV were obtained using a cellular system with codon-modified HPV capsid genes [34]. Briefly, HPV 58 L1 and L2 genes were designed to contain the most frequently used codons found in highly expressed genes in *Homo sapiens *(FN178626 and FN178627, respectively). L1 and L2 genes were cloned into the mammalian bicistronic expression vector, pIRES (BDBiosciences, Clontech). The HPV58 L1 gene was cloned between the *Nhe*I and *EcoR*I restriction sites of MCS A downstream from the CMV IE promoter. The HPV58 L2 gene was subsequently cloned between the *Xba*I and *Not*I restriction sites of MCS B of pIRES-HPV58 L1 to generate pIRES-HPV58 L1/L2 plasmids of 9.1 kbp. Plasmids of this size were previously shown not to be packaged when forming PsV in a cellular system [35]. DNA plasmid pIRES L2 ΔNLS (7.4 kbp) used for the production of PsV was prepared by classical phenol/chloroform DNA preparation. This plasmid contains the DNA sequence encoding amino acids 12 to 442 of the HPV31 L2 between the *Xba*I and *Not*I restriction sites. This sequence was PCR-amplified from a plasmid containing a *Homo sapiens *codon-adapted full length HPV31 L2 gene [36]. This deleted mutant of the L2 gene was selected to reduce the amount of HPV31 L2 protein exported to the nucleus and to prevent its incorporation into the HPV58 PsV structure. For the generation of HPV58 PsV in 293FT cells, cells were transfected with 0.5 μg DNA, 0.25 μg pIRES HPV31 L2 ΔNLS or 0.25 μg pCMV-GFP, 0.25 μg of pIRES-HPV58 L1/L2 and 1 μl Fugene6 (Roche) per cm^2 ^of the culture area. Cells were harvested two days post-transfection, and PsV were purified as previously described [36] and stored at -80°C until use. Pseudovirions were quantified by Western blotting using CamVir-1 antibody by comparison with known concentrations of HPV58 L1/L2 VLPs. Pseudovirions containing HEV ORF2_108-660 _(PsV31-HEV) were produced using the same procedure as described for HPV 58 PsV using previously described pIRES-HPV31 L1/L2 [36] and pcDNA3 HEV ORF2_108-660_, plasmids [29].

### Immunization protocol

Six-week-old female BALB/c mice (CERJ Janvier, Le Genest St Isle, France) were intramuscularly immunized with the different vaccine preparations. Mice from group 1 received saline, mice from groups 2 and 3 received 1 and 10 μg of pIRES-HPV31 L2ΔNLS plasmid (DNA L2), respectively (Table 1). Mice from groups 4 and 5 received HPV31 L1 and HPV31 L1/L2 VLPs (31 L1L2 VLPs), respectively. Mice from group 6 received 10 μg of HPV31 L1/L2 PsV containing HEV ORF2_108-660 _expression plasmid (PsV31-HEV) [29]. Mice from groups 7 and 8 received HPV58 L1/L2 PsV containing GFP expression plasmid (PsV58-GFP) and HPV58 PsV packaged with HPV31L2ΔNLS plasmid (PsV58-31L2), respectively. In order to eliminate variations in the pseudovirion DNA content, the preparations used were from the same batch. Mice were immunized at days 0, 7 and 21. Two weeks after the last injection, serum samples were collected and stored at -20°C. All animal procedures were performed according to approved protocols and in accordance with the recommendations for the proper use and care of laboratory animals, and experiments were approved by the regional animal ethics commmittee (CREEA Centre-Limousin).

**Table 1:** Composition of the vaccines preparations used and anti-HPV16, HPV18, HPV31 and HPV58 neutralizing antibody titers in mice immunized with the different vaccines.

Group N°		Proteins		Gene	Neutralizing titers			
	Name	L1	L2		HPV16	HPV18	HPV31	HPV58
1	Saline	-	-	-	-	-	-	-

2	DNA L2 (1 μg)	-	-	HPV31 L2Δ	-	-	-	-

3	DNA L2 (10 μg)	-	-	HPV31 L2Δ	-	-	-	-

4	31 L1 VLPs	31	-	-	-	-	2,800	-

5	31 L1L2 VLPs	31	31	-	-	-	3,400	**65**

6	PsV31-HEV	31	31	HEV ORF2	-	-	5,198	**54**

7	PsV58-GFP	58	58	GFP	-	-	**50**	4,650

8	PsV58-31L2	58	58	HPV31 L2Δ	**60**	**400**	**733**	5,382

Cross-neutralizing titers are in bold-faced characters.

### Expression of L2SA and detection of anti- L2 antibodies

L2 protein was expressed in insect cells as a fusion protein. In order to purify the L2 protein from insect cells, the Streptactin (SA) coding sequence [37] including upstream (*Bam*HI and *Sal*I) and downstream (*Hin*dIII) restriction sites was synthesized by Geneart (Regensburg, Germany) using an adapted codon usage for expression in *Spodoptera frugiperda*. The SA sequence was cloned between *Sal*I and *Hin*dIII sites of the pFastBacDual expression vector (Invitrogen) in order to obtain the pFastBacDual SA plasmid. The HPV16 L2 ORF was then fused at the 5' end of the SA ORF. For this purpose, the HPV16 L2ΔNLS ORF (amino acids 12 to 442) was amplified by PCR from a plasmid containing a *Homo sapiens *codon adapted version of the wild type L2 gene (FN297862) using HPV16 L2 F (CCGGATCCGCCACCATGGCCAGCGCCACCCAGCTG) and HPV16 L2Δ R (GTCGACCATGTAGTAGCTGGGGTGCAGGATG). A forward primer was designed to introduce a *Bam*HI site, and a Kozak sequence upstream from the start codon and the reverse primer contained a *Sal*I restriction site. The PCR product was then cloned by TA cloning into the pCR2.1 vector (Invitrogen). Both pCR2.1-16 L2ΔNLS and pFastBacDual SA plasmids were submitted to restriction with *Bam*HI and *Sal*I, and the L2 gene was fused to the Streptactin gene in order to generate the pFastBacDual-16 L2ΔNLS (pFBD-L2SA).

A recombinant baculovirus encoding L2SA was generated using the Bac-to-Bac system (Invitrogen) according to the manufacturer's recommendations. Sf21 insect cells were grown at 27°C in SF900II medium supplemented with penicillin, streptomycin and amphotericin B (Invitrogen). Cells were infected at a m.o.i. of ten and grown for four days. Cells were scraped off, centrifuged at 300 × g and then resuspended in PBS 1× containing 0.5% Nonidet P40 and an anti-protease cocktail (Roche, Meylan, France) and incubated on ice for 30 min. The lysate was centrifuged at 4°C for 10 min at 12,000 × g. The pellet, representing the nuclear fraction, was subjected to sonification (3 × 15 s bursts, Vibracell, Fischer Scientific, France). L2SA protein was purified by affinity on immobilized iminobiotin according to the manufacturer's instructions (Pierce, Ozyme, Montigny le Bretonneux, France).

Two hundred nanograms of L2SA were distributed in half of the wells of a 96-well plate (Maxisorp, Nunc, ATGC, Marne-la-Vallée, France) and incubated at 4°C overnight. After two washes with PBS-Tween (0.1%), the wells were saturated with PBS supplemented with 1% FCS for 1 h at 37°C. Duplicate wells (one test and one control) were incubated with two-fold dilutions (starting at 1:25) of mice sera in dilution buffer (PBS 5×, 1% Tween, 10% FCS) for 1 h at 45°C. After four washes, peroxidase-conjugated goat anti-mouse IgG (Fc-specific) (Sigma Aldrich) diluted 1:1,000 in PBS - Tween (1%) - FCS (10%) was added to the wells and incubated for 1 h at 45°C. After four washes, 0.4 mg/ml o-phenylene-diamine and 0.03% hydrogen peroxide in 25 mM sodium citrate and 50 mM Na_2_HPO_4 _were added. After 30 min, the reaction was stopped with H_2_SO_4 _4N and optical density (OD) was read at 492 nm. For data analysis, OD values obtained in the absence of L2SA were subtracted from OD values of test antigens. A result was considered positive when the difference in OD between test and control wells was greater than 0.2. Individual titers represented the reciprocal of the last dilution giving an OD difference greater than 0.2. Values for individual mice were the means of duplicates. Geometric mean titers (GMTs) were calculated for each group. Animals without detectable antibody titers (< 25) were assigned a titer of 1 for calculation of GMTs.

### Detection of anti-HPV neutralizing antibodies

Neutralization assays were performed by inhibition of pseudoinfection of COS-7 cells by pseudovirions containing the pGL3-luc plasmid (Promega, Charbonnières-les-Bains, France). HPV16 and 18 PsV were produced by the previously published disassembly-reassembly method [38] with some modifications [39]. L1/L2 VLPs (100 μg) were incubated in 50 mM Tris-HCl buffer (pH 7.5) containing 20 mM DTT and 1 mM EGTA for 30 min at room temperature. At this stage, pGL3-luc (10 μg) was added to the disrupted VLPs. The preparation was then diluted with increasing concentrations of CaCl_2 _(up to a final concentration of 5 mM) in the presence of 10 nM ZnCl_2_. Pseudovirions were then dialyzed overnight against PBS 1× and stored at 4°C before use. HPV31 and 58 PsV were obtained using a cellular system with codon-modified HPV capsid genes and pGL3-luc plasmid as described above for HPV58 pseudovirons encoding L2.

COS-7 cells (10^4^/well) were seeded in 96-well plates (TPP, ATGC). After 24 h incubation at 37°C, cells were washed twice before addition of pseudovirion/sera mixture. The amount of pseudovirions was adjusted to obtain a relative luciferase activity of 0.2 RLU (Relative Light Unit) (final dilutions in test wells: 1:500 for HPV16, 1:50 for HPV 18, 1:800 for HPV31, and 1:10,000 for HPV 58). Mock transduced COS-7 cells exhibit 0.00001 RLU (Luminoskan Ascent, Thermo scientific, Courtaboeuf, France). Fifty μl of diluted pseudovirions were mixed with 50 μl of mice sera diluted by two-fold dilution in incomplete DMEM from 1:12.5 to 1:25,600 in order to obtain final serum dilutions of 1:25 to 1:51,200. After 1 h incubation at 37°C, the mixture was added to the wells and plates were incubated 3 h at 37°C. Then 100 μl of complete DMEM were added, and the luciferase gene expression was measured after incubation for 48 h at 37°C (Firefly luciferase 1-step assay kit, Fluoprobes, Interchim, Montluçon, France). The results were expressed as the percentage of inhibition of luciferase activity [36]. The data presented are the means of 2 to 3 determinations performed in duplicate. Neutralization titers were defined as the reciprocal of the highest dilution of mice sera that induced at least 50% reduction in luciferase activity. Geometric mean titers were calculated for each group. Animals without detectable neutralizing antibodies were assigned a titer of 1 for the calculation of GMTs.

### Statistical analysis

Geometric mean titers were compared to evaluate ELISA and neutralizing responses. Group results (10 animals per group) were compared by Student *t *test using XLStat software (Addinsoft, Paris, France).

## Results

### Production of HPV58 pseudovirions

**Figure 1:**
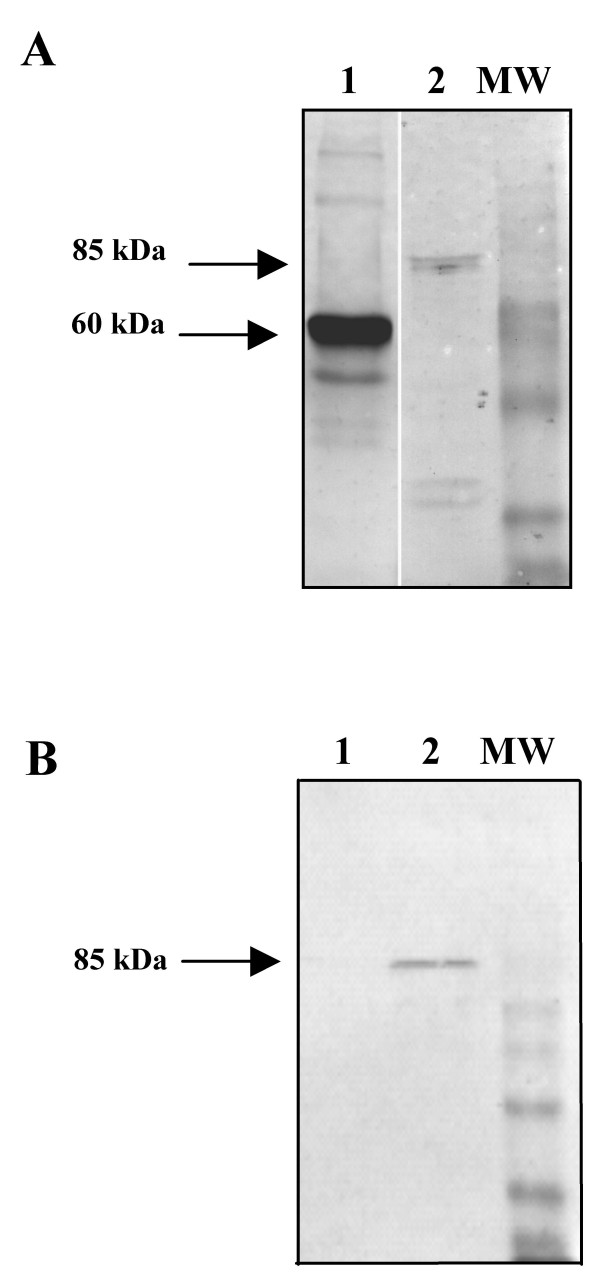
**Western blot**. **A/**Analysis by Western blotting of the HPV58 pseudovirion capsid proteins. L1 was detected using the CamVir-1 monoclonal anti body (lane 1). L2 was detected using polyclonal anti-HPV16 L2 rabbit antiserum (lane 2). **B/**Detection of L2 protein by Western blotting using polyclonal anti-HPV16 L2 rabbit antiserum.Cos-7 cells were transduced with HPV58 pseudovirions encoding GFP (lane 1) or with HPV58 pseudovirion encoding HPV31 L2 (lane 2).

In order to generate HPV58 PsV, 293FT cells were transfected simultaneously with the pIRES-HPV58 L1/L2 plasmid encoding the structural proteins of HPV58 and the pGL3 plasmid encoding luciferase. Three days post-transfection, the nuclear fraction of 293FT cells was analysed by Western blotting. HPV58 L1 and L2 proteins were efficiently expressed (Fig. 1A). Then the ability of PsV58-31L2 to transduce the HPV31 L2 ΔNLS gene was investigated by pseudo-infection of COS-7 cells. Western Blot analysis of L2 protein expression indicated that L2 was detected two days after transduction (Fig. 1B). In order to rule out the possibility that L2 detected in COS-7 cells was due to the presence of the HPV58 L2 contained in the pseudovirion structure, COS-7 cells were transduced with similar PsV packaged with the GFP gene. The presence of L2 was not evidenced in the latter condition (Fig. 1B).

After purification, samples of HPV58 PsV stock were titered by measuring their end-point luciferase gene transduction capacities on Cos-7 cells, and compared with HPV31 PsV obtained in the same cellular system and experimental conditions. Using endpoint titers with a cut-off based on the background luminescence of mock transduced Cos-7 cells, HPV 58 L1/L2 PsV were shown to be 20 times more efficient than HPV31 L1/L2 (data not shown). In view of this result, HPV58 L1/L2 PsV were selected to develop pseudovirion-based immunization.

### Anti-HPV16-L2 immune response in mice immunized with heterologous VLPs and pseudovirions

Anti-HPV16 L2 antibodies were not detected in non-immunized mice (group 1). Anti-L2 antibodies were not detected in mice immunized with HPV31 L1 VLPs (group 4), but were detected in all mice immunized with the LIL2 VLPs (group 5), with a GMT of 1,100.

Anti-L2 antibodies were detected at similar levels in mice immunized with control PsV (groups 6 and 7), with GMTs of 855 and 1,212 (p = 0.459). By comparison with these control pseudovirions, the anti-L2 GMT (2,600) was higher in mice immunized with PsV58-31L2 (p = 0.001 and p = 0.101, respectively).

### Induction of cross-neutralizing antibodies

**Figure 2:**
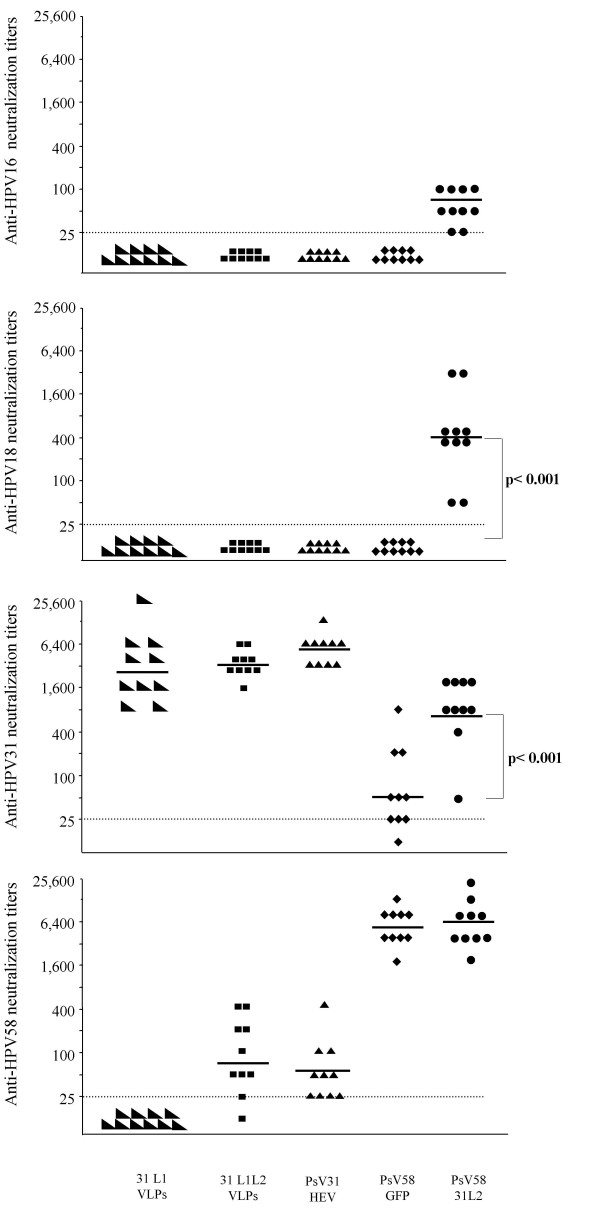
**Detection of HPV16, HPV18, HPV31 and HPV58 neutralizing antibodies**. The individual mouse neutralizing titers are the means of the last reciprocal dilution providing more than 50% inhibition of luciferase expression. Animals without detectable antibody titers (< 25, dotted line) were assigned a titer of 1 for calculation of GMTs (horizontal bars).

Homologous HPV31 neutralizing antibodies were detected in mice immunized with HPV31 L1 or HPV31 L1L2 VLPs and HPV31 HEV PsV (groups 4, 5 and 6), with GMTs of 2,800 ± 2360, 3,400 ± 460 and 5,198 ± 900, respectively (GMT ± SEM). Low titers of HPV58 neutralizing antibodies were only observed in mice receiving HPV31 L1L2 VLPs (group 5) and HPV31 PsV containing the HEV ORF2 irrelevant gene (group 6). No neutralizing antibodies against HPV16 and HPV18 were detected in any of the mice from groups 4 to 6 receiving HPV31 VLP vaccine preparations (Fig. 2).

High levels of homologous neutralizing antibodies were detected in mice immunized with HPV58 PsV (groups 7 and 8), with GMTs of 4,650 ± 980 and 5,382 ± 2240, respectively. Low levels of neutralizing antibodies to HPV31 (GMT = 50 ± 315) were detected in mice immunized with PsV58-GFP, and a dramatic increase in anti-HPV31 neutralizing antibodies (with a GMT of 733 ± 190) was observed in mice immunized with PsV58-31L2. Moreover, neutralizing antibodies against HPV16 and HPV18 were only detected in mice immunized with the PsV58-31L2, with GMTs of 60 and 400, respectively (Table 1).

## Discussion

Since no differences in antibody titers or in protection were observed in animal studies [40] when immunization with L1 and L1/L2 VLPs were compared, it was generally believed that there was insufficient reason to introduce L2 protein into the composition of VLPs. In addition, L2 protein assembled in L1 VLPs is weakly immunogenic due to the immunodominance of L1 [23]. However, our findings suggested that even in the absence of adjuvant cross-neutralizing antibodies could be obtained by incorporating L2 in the composition of the VLPs (group 5) or pseudovirions encoding irrelevant genes (groups 6-7) compared to L1 VLPs (group 4), despite the low anti-L2 immune response (GMT 855 to 1212). Anti-L2 antibody titers are generally several orders of magnitude lower than the anti-L1 titers obtained with VLP vaccines. However, even low anti-L2 antibody levels have been shown to be sufficient for protection [22,26], this being in part explained by the slow uptake kinetics into cells reported for HPVs [41]. In addition, we evaluated the immune response obtained in mice immunized with 10 μg of L2SA fusion protein without adjuvant. In these mice, L2 protein induced only a weak anti-HPV16 L2 response (GMT = 348), and a weak homologous neutralizing response in 3 out of 10 mice. Cross-neutralizing antibodies to HPV 18, 31 and 58 were not detected. These results differ from previously published results [23] in which broad spectrum cross-neutralization was observed in rabbits immunized with higher doses of L2 protein (100 μg) in combination with Freund's adjuvant. The induction of higher levels of cross-neutralization of HPV 31 L1/L2 VLPs compared to HPV 31 L1 VLPs suggested that, due to the potential antigenic competitions, HPV L1/L2 VLP of a limited number of genotypes would be a much easier solution compared to the technical complexity of generating a multivalent vaccine [42].

Since HPV16 and HPV18 PsV and HPV31 and HPV58 PsV were produced in different ways, with different infection titers and particle-to-infectivity ratios, the results obtained might have been affected by the fact that the different neutralization assays might not have the same sensitivity. The HPV16 neutralization assay performed with PsV produced by the dissociation reassociation method [39] appeared to be less sensitive than HPV 31 and 58 neutralization assays performed with PsV obtained in mammalian cells. We therefore investigated the relative sensitivity of the assays by comparing the ratio between homologous neutralizing titers and homologous ELISA titers for each type. These ratios were 0.22, 0.93, and 0.71 for HPV 16, 31, 58, respectively, indicating that the HPV16 neutralizing assay is 3.5 less sensitive than the HPV58 neutralizing assay and 4.2 less sensitive than the HPV31 neutralizing assay. These differences in sensitivity may explain why HPV16 neutralizing antibodies were not detected in mice immunized with HPV31 (groups 5 and 6) for which HPV58 neutralizing titers of 65 and 54 were observed. This also explains the low HPV16 neutralizing titers observed in mice immunized with PsV58-31L2 (group 8) compared to those of HPV18 and 31. Although the intensity of cross-neutralizing responses was not directly comparable to other studies, our findings clearly indicate that the highest levels of cross-neutralizing antibodies were observed with PsV encoding the HPV31 L2 protein. However, the ratio of neutralizing antibody titers against heterologous types to those against homologous types represented 1% in mice immunized with L1L2 VLPs or control PsV, whereas a ratio of around 10% was observed in mice immunized with PsV encoding the HPV31 L2 protein. The latter ratio is in agreement with those reported by Gambhira et al [25] and Alphs et al [26] using L2 peptides and potent adjuvants.

The *de novo *synthesis of HPV 31 L2 from the L2 gene packaged in HPV58 PsV is likely to have a critical role in the induction of cross-neutralization, since neutralizing antibodies against HPV16 and a more genetically distant type from the alpha-7 clade (HPV18) were only detected in mice immunized with the HPV58 PsV encoding L2 (group 8) and not in mice immunized with HPV58 PsV encoding GFP (group 7). In addition, the higher anti-HPV31 neutralizing titers observed in mice from group 8 (GMT = 733) was likely to have been due to the *de novo *production of L2 protein due to the transduction of the HPV31 L2 plasmid, since the mice from group 7 immunized with PsV GFP presented a GMT of only 50 (p < 0.001). This was correlated to the fact that the highest anti-HPV16 L2 antibody titers observed in mice from group 8 were associated with the highest and broadest detection of cross-neutralizing antibodies.

As the HPV31 L2 protein encoded by the pIRES HPV31 L2 ΔNLS plasmid may be part of the HPV 58 PsV structure, this HPV31 L2 might have a role in the cross-neutralizing response. The HPV 31 L2 protein without N- and C-terminus NLS sequences was expected not to reach the nucleus where pseudovirions are assembled. In fact, HPV31 L2 protein was still detected in the nuclear fraction of producer cells (data not shown), in agreement with previous reports by [43]. Moreover, it was not possible to differentiate between the presence of HPV31 and HPV58 L2 in the capsid. However, the deleted HPV31 L2 should be excluded from the pseudovirion capsid since the C-terminus NLS has been shown to be necessary for *in vivo *interaction between L2 and L1 in the BPV-1 model [44].

It's possible that the third injection of pseudovirions was not necessary in mice immunized with PsV58-31L2 since it could be expected that the first two injections would have induced anti-HPV58 neutralizing antibodies that would block the expression of the HPV31 L2 protein. In order to investigate this, sera were obtained one week after the second injection from these mice and then tested for the presence of neutralizing antibodies against HPV16 and 31. Before the booster, anti-HPV31 neutralizing antibodies were detected at a GMT of 77, and this rose to 733 after the booster dose. HPV16 neutralizing antibodies were not detected after the second dose but reached a GMT of 50 after the booster. This booster effect was probably due to a response to the *de novo *expressed HPV31 L2 protein and was not a booster effect due to the immune response to L1 and L2 proteins from the pseudovirion capsid, since a cross-neutralizing antibody titer of only 50 was observed in mice immunized with PsV58-GFP in comparison with a GMT of 733 in mice immunized with PsV58-31L2.

## Conclusions

HPV58 PsV encoding the HPV31 L2 protein were produced in order to develop a vaccine with the potential to protect against a broad spectrum of high-risk HPV types, and their capacity to induce cross-neutralizing antibodies was investigated in mice. The findings confirmed that L2 protein assembled into VLPs is less immunogenic than L1 and that L1 plus L2 VLPs induced more cross-neutralizing antibodies than L1 alone assembled into VLPs, and indicated that high levels of cross-neutralizing antibodies are only obtained after immunization with pseudovirions encoding the L2 protein. The addition of an adjuvant is however essential to achieve levels of cross-protective antibodies similar to the levels of neutralizing antibodies observed with the current L1 vaccines. L2-pseudovirions are a promising strategy in the development of broader-spectrum HPV vaccines in addition to chimeric L1-L2 VLPs or L2 peptide formulations [30,26].

## Competing interests

Patent for pseudovirions with Aurabiosciences.

## Authors' contributions

NC produced the HPV58 PsV, participated in the production of VLPs, the detection of neutralizing antibodies and immunization studies and helped to draft the Manuscript, MF produced the HPV31 PsV, contributed to the detection of neutralizing antibodies and helped to draft the manuscript. ES, TR, JG, and DFDF participated in the production of VLPs, the detection of neutralizing antibodies and immunization studies. AT and PC conceived the study, participated in its design and coordination and helped to draft the manuscript. All authors have read and approved the final manuscript.
